# Supplementary material for: A cryptic third active site in cyanophycin synthetase creates primers for polymerization
Source: Nat Commun. 2022 Jul 7;13:3923. doi: 10.1038/s41467-022-31542-7 (PMC9262961; doi:10.1038/s41467-022-31542-7)
Supplement: Supplementary file 1 — Supplementary information [file 41467_2022_31542_MOESM1_ESM.pdf]

A cryptic third active site in cyanophycin synthetase creates primers for polymerization

Supplementary information

Itai Sharon[1], Sharon Pinus[2], Marcel Grogg[3], Nicolas Moitessier[2], Donald Hilvert[3] & T. Martin Schmeing[1]

[1]Department of Biochemistry and Centre de recherche en biologie structurale, McGill University, Montréal, QC, Canada, H3G 0B1.

[2]Department of Chemistry, McGill University, 801 Sherbrooke St W, Montreal, QC, Canada H3A 0B8.

[3]Laboratory of Organic Chemistry, ETH Zürich, CH-8093 Zürich, Switzerland.

Correspondence e-mail: martin.schmeing@mcgill.ca

SUPPLEMENTARY TABLES

Supplementary Table 1. Cryo-EM data collection, refinement and validation statistics.

	*Su*CphA1 E82Q + ATP + (β-Asp-Arg)$_{16}$ (EMD-26161) (PDB 7TXV)	*Su*CphA1 WT + ATP + (β-Asp-Arg)$_{16}$ (EMDB-23326*) (PDB 7TXU)
**Data collection and processing**		
Magnification	105,000x	
Voltage (kV)	300	
Electron exposure (e–/Å^2)	60	
Defocus range (μm)	-1.0 to -2.0	
Pixel size (Å)	0.855	
Symmetry imposed	D2	
Initial particle images (no.)		
Final particle images (no.)	318,594	
Map resolution (Å)<br>FSC threshold 0.143	2.7	
Map resolution range (Å)	2.4-5.5	
**Refinement**		
Model resolution (Å)<br>FSC threshold 0.143	2.7	2.6
Model resolution range (Å)	2.4-5.5	2.4-9.0
Map sharpening *B* factor (Å^2)	-106	-105
Model composition		
Non-hydrogen atoms	27656	27408
Protein residues	26584	26584
Ligands	1072	824
B factors (Å^2)		
Protein	70	45
Ligand	61	40
R.m.s. deviations		
Bond lengths (Å)	0.022	0.020
Bond angles (°)	1.959	1.913
Validation		
MolProbity score	1.34	1.28
Clashscore	2.57	1.81
Poor rotamers (%)	0.70	0.14
Ramachandran plot		
Favored (%)	95.73	95.03
Allowed (%)	4.16	4.97
Disallowed (%)	0.12	0.0

*The collection statistics for EMDB-23326 were previously reported[1].

Supplementary Table 2. ICP-MS analysis of *Su*CphA1, *Su*CphA1 N domain and a buffer control. All units are in µg/kg. BQ = below limit of quantitation.

Sample	55Mn	56Fe	59Co	60Ni	65Cu	66Zn
**Buffer control**	BQ < 0.88	BQ < 26.1	BQ < 0.46	BQ < 2.05	4.48	BQ < 6.85
***Su*CphA1**	1.14	44.2	BQ < 0.46	424	989	5382
***Su* N domain**	BQ < 0.89	49.2	0.77	1501	322	3747
***Tm*CphA1**	BQ < 1.03	108	BQ < 0.70	82.7	218	109

Supplementary Table 3. DNA primers used in this study for cloning.

Sequence	Name
CATATGTTTTACCTCCTTAAAAGTTAAAC	R. pBacIT
GAGAATTTGTACTTCCAAGGTCACC	F. pBacPtandem reverse2
TTAACTTTTAAGGAGGTAAAAACATATGaaaattcttaaaactctgactctccg	F. UTEX2470 cphA into pBacIT
TGGTGACCTTGGAAGTACAAATTCTCaccaatgggattgcggacc	R. UTEX2470 cphA into pBacIT
gattaaacccctggatggcaacGCtggccggggcatcacc	F. UTEX2470 H267A
ggtgatgccccggccaGCgttgccatccaggggtttaatc	R. UTEX2470 H267A
ctacctgtcaattttggagggttccGCgactctgcgggtggagcag	F. UTEX2470 W672A
ctgctccacccgcagagtcGCggaaccctccaaaattgacaggtag	R. UTEX2470 W672A
gctgaatgtggcggcggCtGCcctggggctgggggatattg	F. UTEX2470 D585A H586A
caatatcccccagccccaggGCaGccgccgccacattcagc	R. UTEX2470 D585A H586A
GTTAGTAGCAGCAGCGCGCCGTGTGccagcccatgtgactgg	F. UTEX2470 omega into DSM23827
TAACATCTACAGCGGTCCCACCTGTgcttaggttagcggtggc	R. UTEX2470 omega into DSM23827
GGTGGGACCGCTGTAGATG	F. DSM23827 change omega
ACGGCGCGCTGCTGC	R. DSM23827 change omega
GGGCGATCTCGGTCTTCCTGTACCTcggggcaccaccatcc	F. 2470 lid into 23827
CGTTGACAACCAGTAAGCGATGGTCactgccttcgtagtaacgctc	R. 2470 lid into 23827
GACCATCGCTTACTGGTTG	F. 23827 change lid
AGGTACAGGAAGACCGAG	R. 23827 change lid
TCACTAATTCCATCGCAGACGGCCCcagggccgagttagcc	R. UTEX2470 CphA N into 23827 CphA
GGGCCGTCTGCGATGG	F. DSM23827 CphA change N
GATCCGCTCAGGATTACGCACGTTTaccacatcagcagaacag	F. UTEX2470 Mlid into 23827 CphA
GGTGACCTTGGAAGTACAAATTCTCaccaatgggattgcgg	R. UTEX2470 Mlid into 23827 CphA
AAACGTGCGTAATCCTGAG	R. 23827 CphA change Mlid
cttatatgggtGCtattgtcgagc	F. UTEX2470 CphA1 H79A
gctcgacaataGCacccatataag	R. UTEX2470 CphA1 H79A
gtcatattgtcgCgGCtgtggccctgg	F. UTEX2470 E82A H83A
ccagggccacaGCcGcgacaatatgac	R. UTEX2470 E82A H83A
ggtcatattgtcCagcatgtggc	F. UTEX2470 E82Q
gccacatgctGgacaatatgacc	R. UTEX2470 E82Q
GGTGACCTTGGAAGTACAAATTCTCtaaatcccgcaaatcttcc	R. UTEX2470 N domain
ggccctaactCttggagtaCtcgacgcaag	F. UTEX2470 Y14S I17T
cttgcgtcgaGtactccaaGagttagggcc	R. UTEX2470 Y14S I17T
ggaacacttcGCctcgccggg	F. UTEX2470 C59A

cccggcgagGCgaagtgttcc	R. UTEX2470 C59A
gggttttggcGCcaccagggaaac	F. UTEX2470 R100A
gtttccctggtgGCgccaaaaccc	R. UTEX2470 R100A
ggatttttggaaGCggtaaaagaaggg	F. UTEX2470 R70A
cccttcttttaccGCttccaaaaatcc	R. UTEX2470 R70A
ccctggtggaaGCcttctgctcgc	F. UTEX2470 H57A
gcgagcagaagGCttccaccaggg	R. UTEX2470 H57A
gggatttttggaaGCggtaaaagaagggac	F. UTEX2470 R70A 2
gtcccttcttttaccGCttccaaaaatccc	R. UTEX2470 R70A 2
cctggtggaaGCcttctgctcg	F. UTEX2470 H57A 2
cgagcagaagGCttccaccagg	R. UTEX2470 H57A 2
aGAAAGGTGACAacACGGGCCCGctgTCGGCTAAAATGGTTCTTCG	F. DSM23827 V516E M520N V524L
cagCGGGCCCGTgtTGTCACCTTTCtCGGTTACATTACCATCGATCAG	R. DSM23827 V516E M520N V524L
GCTGTTGAGATTGCAtCAGATAAGAATATGTG	F. DSM23827 CphA A204S
CACATATTCTTATCTGaTGCAATCTCAACAGC	R. DSM23827 CphA A204S
CGACTTCAGTGTTTcgTGGCCCGAATACG	F. DSM23827 CphA V10R
CGTATTCGGGCCAcgAAACACTGAAGTCG	R. DSM23827 CphA V10R
GGTATCGCATGAATGATcgtTCTCTTATCCAGGTAGG	F. DSM23827 CphA A173R
CCTACCTGGATAAGAGAacgATCATTCATGCGATACC	R. DSM23827 CphA A173R
GGATTGAGGCCaCTTTGACCTC	F. DSM23827 CphA A190T
GAGGTCAAAGtGGCCTCAATCC	R. DSM23827 CphA A190T
GTTGAGATTGCAtgcGATAAGAATATGTG	F. DSM23827 CphA A204C
CACATATTCTTATCgcaTGCAATCTCAAC	R. DSM23827 CphA A204C
GATTAATGCGGcGCCTGGTCTG	F. DSM23827 CphA G437A
CAGACCAGGCgCCGCATTAATC	R. DSM23827 CphA G437A
GGcGCCTGGTtTtCGCATGCATgTCTCTCCGTC	F. DSM23827 L440F I444V
GACGGAGAGAcATGCATGCGaAaACCAGGCgCC	R. DSM23827 L440F I444V

Supplementary Table 4. HR-MS analysis of the cyanophycin segments used in this study.

Molecule	Charge	Expected m/z	Observed m/z
β-Asp-Arg	+1	290.14590	290.14560
(β-Asp-Arg)-Asp	+1	405.17284	405.17276
$(\beta\text{-Asp-Arg})_2$	+1	561.27395	561.27327
$(\beta\text{-Asp-Arg})_3$	+1	832.40066	832.40062
$(\beta\text{-Asp-Arg})_4$	0	1103.53006	1103.52799
$(\beta\text{-Asp-Arg})_8\text{-NH}_2$ (reference [1])	0	2187.05826	2187.06290
$(\beta\text{-Asp-Arg})_8$-Asn (reference [1])	+2	1151.54624	1151.54538
$(\beta\text{-Asp-Arg})_{12}$ (reference [1])	+3	1091.52301	1091.52134

SUPPLEMENTARY FIGURES

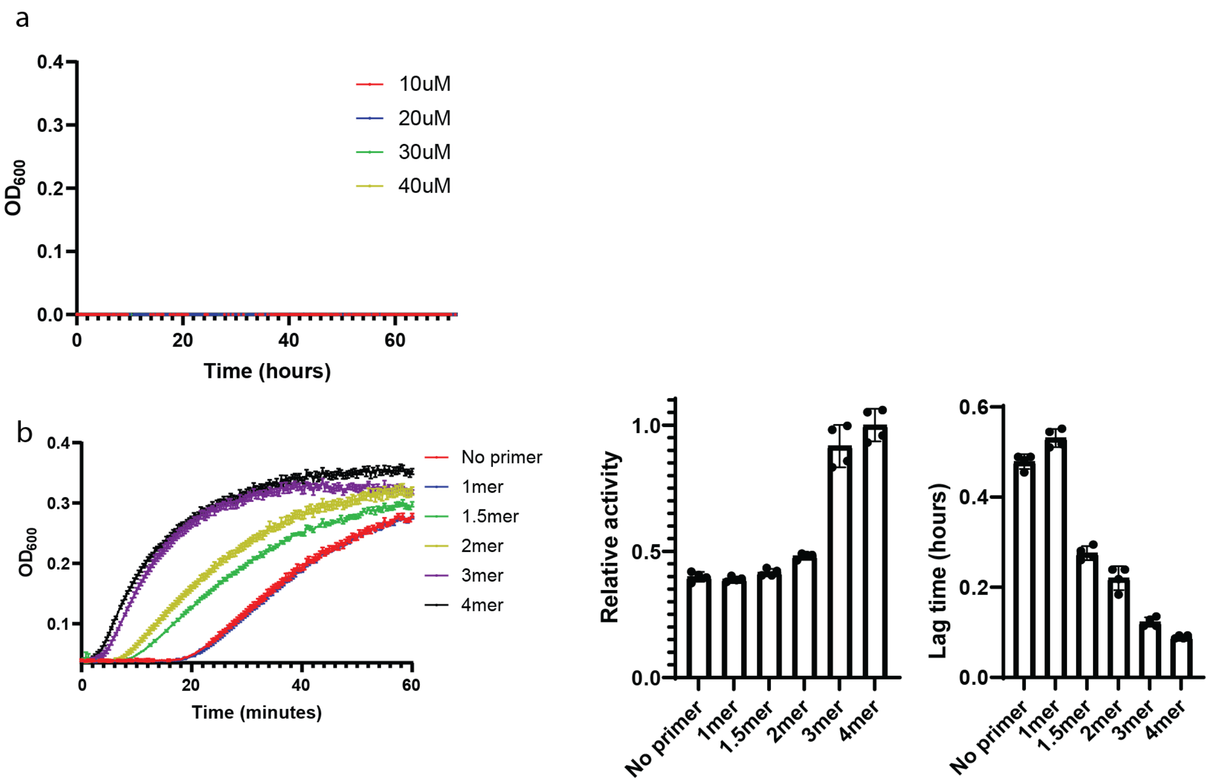

Supplementary Figure 1. *Su*CphA1 primer-dependence. a Activity assay plots of different concentrations of *Tm*CphA1 in the absence of primer. Without primer, the enzyme displays no activity after 3 days, even at high concentrations. n=4 independent experiments. Data are presented as mean value. **b** Activity assay plots, activity rate values and lag time of *Su*CphA1 in the presence of various cyanophycin primers. Asp, Arg and β-Asp-Arg display similar activity profiles, suggesting β-Asp-Arg are not used as primers. (β-Asp-Arg)-Asp and (β-Asp-Arg)$_2$ both shorten the lag phase before onset of activity, suggesting they are moderately good primers. (β-Asp-Arg)$_3$ and (β-Asp-Arg)$_4$ are both equally good primers for this enzyme. n=4 independent experiments. Data are presented as individual measurements and mean value, error bars represent SD values.

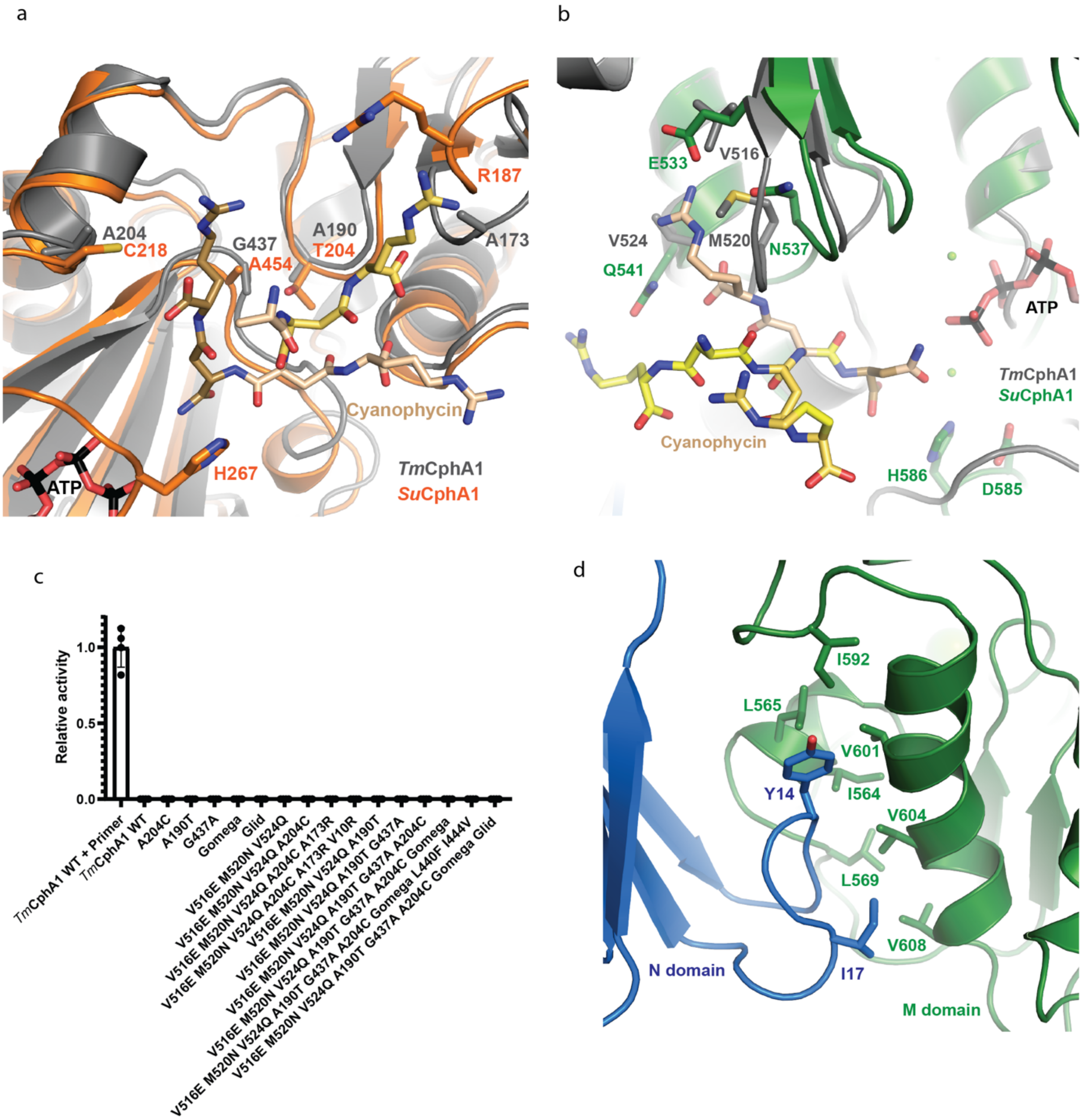

Supplementary Figure 2. CphA1 mutations. a Overlay of *Su*CphA1 (orange) and *Tm*CphA1 (gray) G domain active sites. Polymer binding residues which are different in these enzymes are labeled. **b** Overlay of *Su*CphA1 (green) and *Tm*CphA1 (gray) M domain active sites. Polymer binding residues which are different in these enzymes are labeled. **c** Attempts to mutate *Tm*CphA1 G and M active sites to make them more similar to those of *Su*CphA1 did not result in primer-independent activity, suggesting these active sites are not responsible for primer-independent activity. n=4 independent experiments. Data are presented as individual measurements and mean value, error bars represent SD values. **d** The loop containing *Su*CphA1 Y14 and I17 interacts with a hydrophobic patch on the M domain, thus burying those hydrophobic residues. Double mutations Y14S I17T allowed soluble expression and purification of the excised N domain.

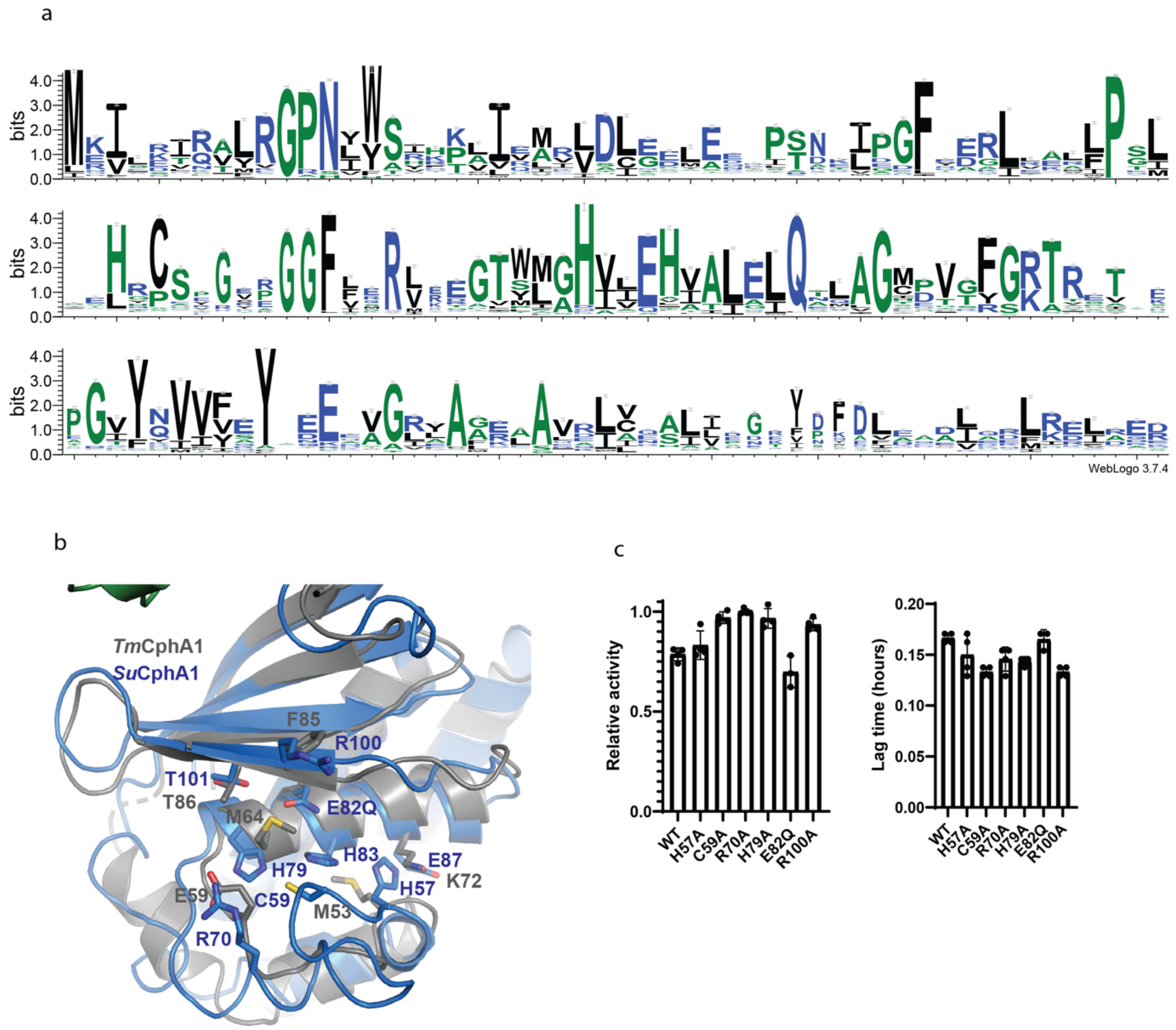

Supplementary Figure 3. Important residues for N domain activity. a Weblogo[2] diagram covering the entire N domain of CphA1. This Weblogo was constructed from sequence alignments of CphA1 enzymes using ClustalW[3], and excludes cyanophycin synthetase 2 (CphA2) sequences. CphA2s are specialized cyanobacterial enzymes that polymerize β-Asp-Arg dipeptides recovered from degraded cyanophycin[4,5]. CphA2 N domains share low sequence identity to CphA1 N domains and the N domain active site motif is absent from CphA2 sequences. **b** Overlay of the N domains of *Tm*CphA1 (PDB code 7LGN [https://www.rcsb.org/structure/7LGN], gray) and *Su*CphA1 (blue). The domains have a similar overall structure, but with key differences in sequence. Conserved residues found in *Su*CphA1 and the residues in equivalent positions in *Tm*CphA1 are labeled. **c** Activity rate and lag time of *Su*CphA1 N domain mutants with $(\beta\text{-Asp-Arg})_3$ as primer. The mutants displayed similar or slightly higher activity rates to those of the WT enzyme. n=4 independent experiments. Data are presented as individual measurements and mean value, error bars represent SD values.

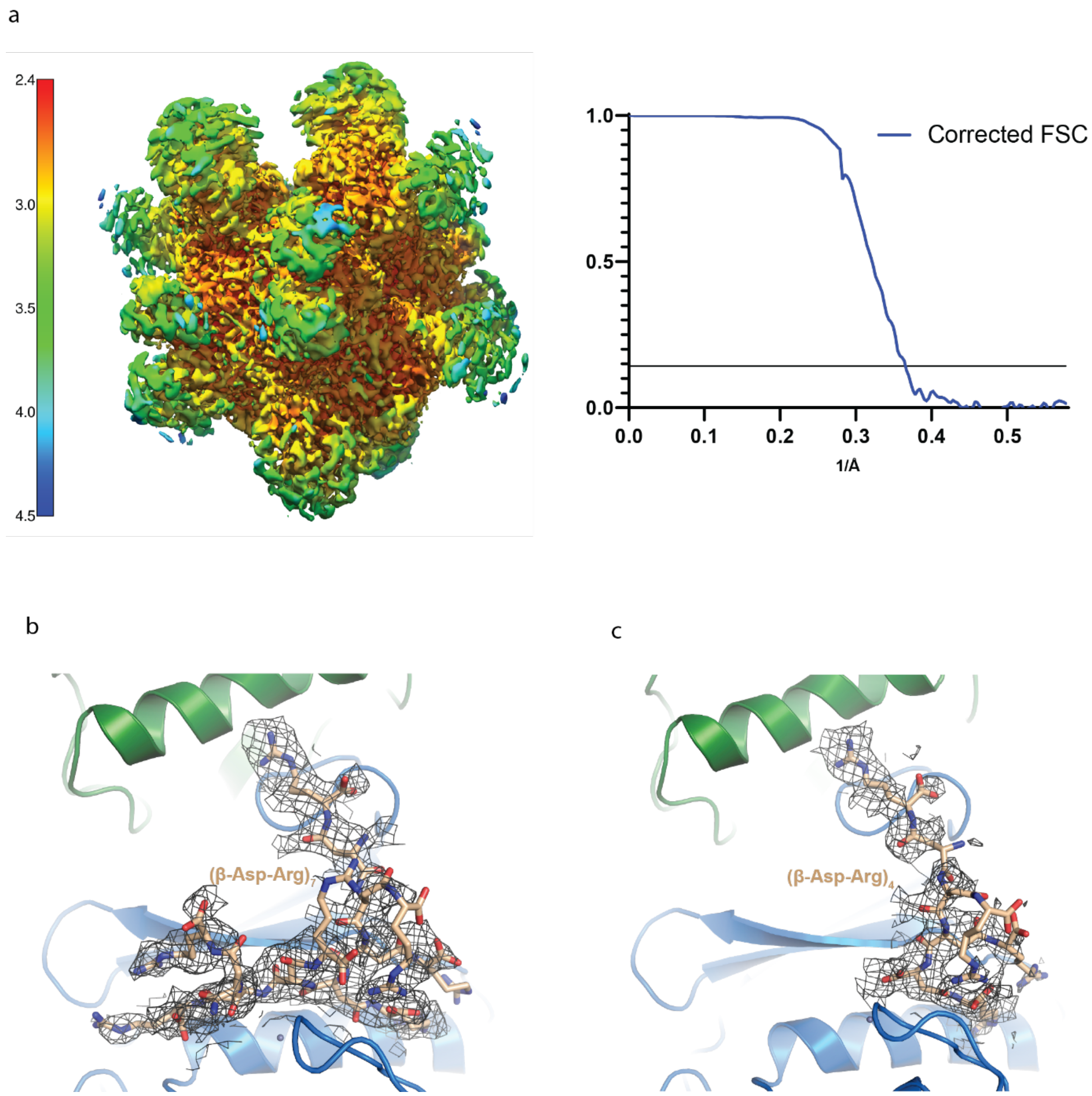

Supplementary Figure 4. Cryo-EM of SuCphA1 with cyanophycin. a The cryo-EM map of *Su*CphA1 E82Q colored by local resolution and the phase-randomized FSC curve of the map. **b** The map of *Su*CphA1 E82Q with ATP and (β-Asp-Arg)$_{16}$, with (β-Asp-Arg)$_{7}$ fit in the map near the N domain active site. The map is displayed at a contour level of 5 with 2.5 Å carving around the ligand. **c** Cryo-EM map[1] of *Su*CphA incubated with (β-Asp-Arg)$_{16}$. Signal for a chain of four dipeptide residues is visible in this map. Similar signal is seen in maps of complexes containing (β-Asp-Arg)$_{8}$-NH_2 or (β-Asp-Arg)$_{8}$-Asn, but not in maps of complexes containing only ATP, Arg and Asp[1]. The map is displayed at a contour levels of 5 with 2.5 Å carving around the ligand.

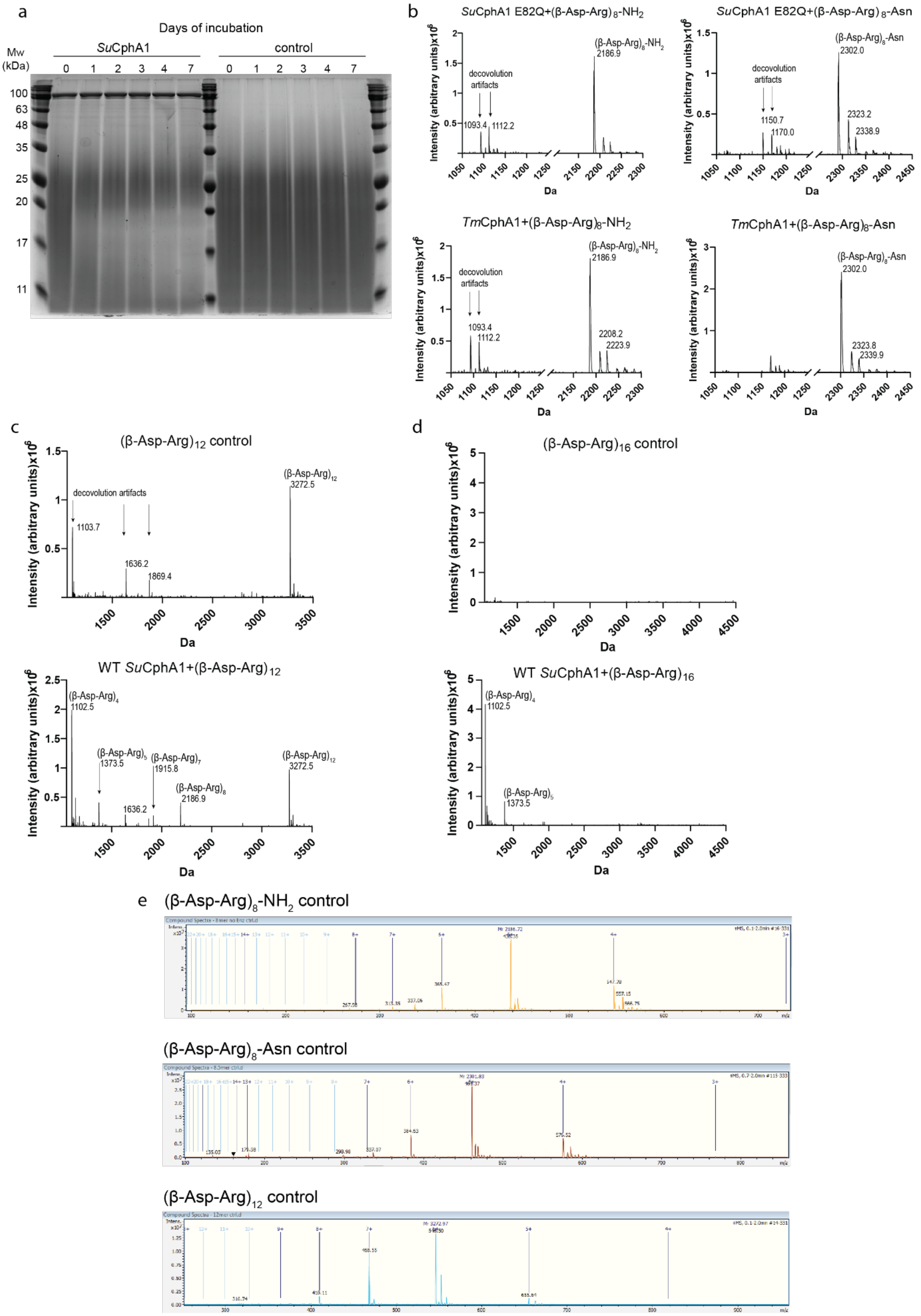

Supplementary Figure 5. Mass spectra of cyanophycin degradation assays. a SDS-PAGE analysis of cyanophycin degradation assays. Polymer purified from *E. coli* expressing *Tm*CphA1 was incubated

with and without *Su*CphA1 over several days. The gradual decrease in smear intensity, especially between the 11 and 20 kDa markers, shows cyanophycin is being slowly degraded in the presence of *Su*CphA1. n=3 independent experiments. The uncropped gel images are presented in **Supplementary Fig. 7**. **b** MS traces of (β-Asp-Arg)$_8$-NH_2 and (β-Asp-Arg)$_8$-Asn after incubation with *Su*CphA1 E82Q or WT *Tm*CphA1. **c** MS traces of (β-Asp-Arg)$_{12}$ before (top) and after (bottom) incubation with WT *Su*CphA1. After incubation with enzyme, the peak matching (β-Asp-Arg)$_{12}$ (expected at 3271.9 Da) is reduced and peaks corresponding to (β-Asp-Arg)$_4$ (expected at 1102.5 Da), (β-Asp-Arg)$_5$ (expected at 1373.6 Da), (β-Asp-Arg)$_7$ (expected at 1915.9 Da) and (β-Asp-Arg)$_8$ (expected at 2187.0 Da) appear. **d** MS traces of (β-Asp-Arg)$_{16}$ before (top) and after (bottom) incubation with WT *Su*CphA1. No signal was observed for (β-Asp-Arg)$_{16}$ under these conditions, presumably because the used MS conditions result in low signal for long cyanophycin chains. After incubation with enzyme, the peak matching (β-Asp-Arg)$_4$ (expected at 1102.5 Da) and (β-Asp-Arg)$_5$ (expected at 1373.6 Da) appear. **e** Representative raw MS spectra of cyanophycin controls and degradation products. The charged-state rulers show that all major peaks can be accounted for. These spectra were deconvoluted to produce the data in figures 4a-c.

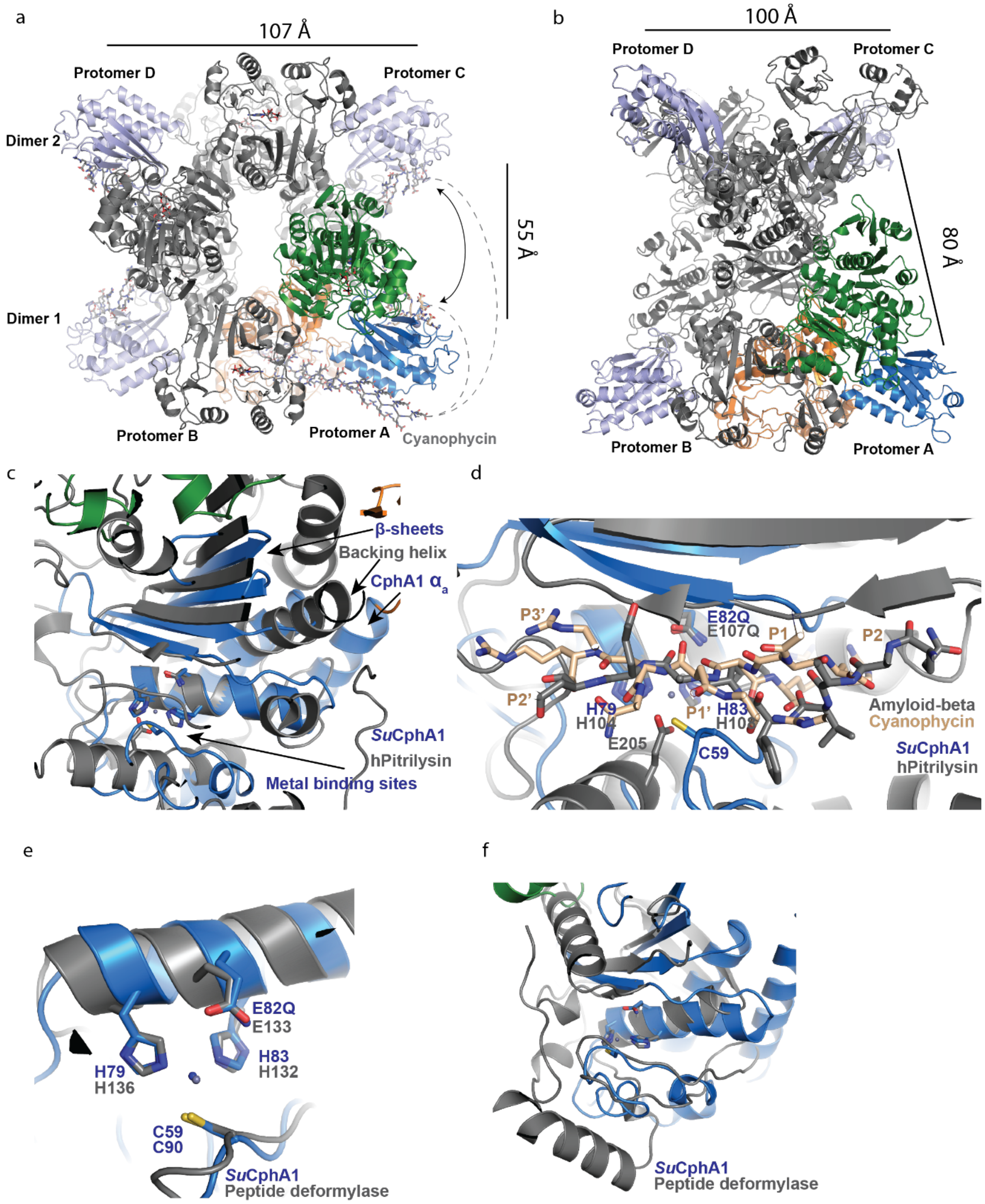

Supplementary Figure 6. N domain orientation in CphA1 tetramers and homology to M16 family members. a *Su*CphA1 with modelled cyanophycin bound to the charged patches on the N domain. Dashed lines show a possible route of cyanophycin to the N domain active sites. The dimer architecture (chains A+B, C+D) positions N domain active sites (measured as the distance between the two Zn ions) 107 Å apart and facing away from each other. In a tetramer, however, N domains from adjacent dimers (chains A+C, B+D) face each other and are only 55 Å apart. **b** The tetramer architecture of *Tm*CphA1 (PDB code 7LGN [https://www.rcsb.org/structure/7LGN]) leads to an increased distance of ~80 Å

between N domains from different dimers (chains A+C, B+D). However, as these N domains lack catalytic activity. **c** Alignment of *Su*CphA1 N domain (blue) and human pitrilysin[6] (PDB code 4NGE [https://www.rcsb.org/structure/4NGE], gray) shows moderate structural conservation of the metal binding site helix (pitrilysin residues 101-113), core β-sheet (pitrilysin residues 138-150, 86-93, 263-269) and backing helix (pitrilysin residues 153-168). **d** Alignment of the active sites of *Su*CphA1 N domain (blue) and human pitrilysin (PDB code 4NGE [https://www.rcsb.org/structure/4NGE], gray) shows structural conservation of the metal binding residues and substrate positioning. **e** Alignment of the active sites of *Su*CphA1 N domain (blue) and *E. coli* peptide deformylase[7] (PDB code 1DFF [https://www.rcsb.org/structure/1DFF], gray) shows high structural similarity of the C-H-H metal binding triad. **f** Alignment of the overall structures of *Su*CphA1 N domain (blue) and *E. coli* peptide deformylase[7] (PDB code 1DFF [https://www.rcsb.org/structure/1DFF], gray) shows the two enzymes share little structural similarity.

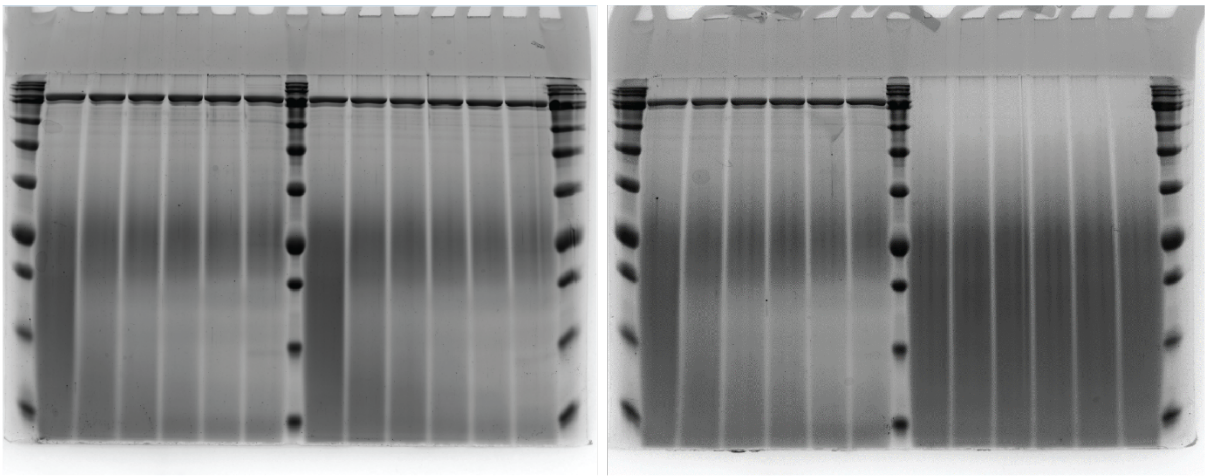

Supplementary Figure 7. Non-cropped gels. Gels associated with supplementary figure 5a.

SUPPLEMENTARY REFERENCES

1. Sharon, I. et al. Structures and function of the amino acid polymerase cyanophycin synthetase. *Nat Chem Biol* 17, 1101–1110 (2021).
2. Crooks, G.E., Hon, G., Chandonia, J.M. & Brenner, S.E. WebLogo: a sequence logo generator. *Genome Res* 14, 1188–90 (2004).
3. Thompson, J.D., Higgins, D.G. & Gibson, T.J. CLUSTAL W: improving the sensitivity of progressive multiple sequence alignment through sequence weighting, position-specific gap penalties and weight matrix choice. *Nucleic Acids Res* 22, 4673–80 (1994).
4. Klemke, F. et al. CphA2 is a novel type of cyanophycin synthetase in N_2-fixing cyanobacteria. *Microbiology* 162, 526–36 (2016).
5. Sharon, I., Grogg, M., Hilvert, D. & Schmeing, T.M. Structure and function of the β-Asp-Arg polymerase cyanophycin synthetase 2. *ACS Chem Biol* 17(2022).
6. King, J.V. et al. Molecular basis of substrate recognition and degradation by human presequence protease. *Structure* 22, 996–1007 (2014).
7. Chan, M.K. et al. Crystal structure of the *Escherichia coli* peptide deformylase. *Biochemistry* 36, 13904-9 (1997).
